# Molecular detection of *Leishmania* spp in *Lutzomyia longipalpis* in the city of Lavras, Minas Gerais, Brazil

**DOI:** 10.1590/1414-431X20198224

**Published:** 2019-08-29

**Authors:** J.C. Castro, L.L. Bueno, T.F. Milagres, F.D. Rêgo, C.M.F. Gontijo, A.P. Peconick, A.J. Andrade, T.A. Barçante, J.M.P. Barçante

**Affiliations:** 1 Programa de Pós Graduação em Parasitologia, Departamento de Parasitologia, Universidade Federal de Minas Gerais, Belo Horizonte, MG, Brasil; 2 Grupo de Estudo em Leishmanioses, FIOCRUZ, Belo Horizonte, MG, Brasil; 3 Programa de Pós Graduação em Ciências Veterinárias, Departamento de Medicina Veterinária, Universidade Federal de Lavras, Lavras, MG, Brasil; 4 Departamento de Patologia Básica, Universidade Federal do Paraná, Curitiba, PR, Brasil; 5 Programa de Pós Graduação em Ciências da Saúde, Departamento de Ciências da Saúde, Universidade Federal de Lavras, Lavras, MG, Brasil

**Keywords:** Leishmaniasis, Sand fly, Epidemiology

## Abstract

Leishmaniasis is a neglected disease that affects a large part of the world population. Knowing the sand fly fauna of a region is of fundamental importance for guiding health surveillance actions related to the prevention and control of leishmaniasis. A total of 86 specimens of sand flies (60 females and 26 males) were collected. Using the classification proposed by Galati (2003), the following species were identified: *Lutzomyia longipalpis* (Lutz & Neiva, 1912), *Migonemyia migonei* (França, 1920), *Evandromyia cortelezzi* (Brethes, 1923), *Ev. sallesi* (Galvão & Coutinho, 1939), *Nyssomyia whitmani* (Atunes & Coutinho, 1939), *Psathyromyia lutziana* (Costa Lima, 1932), *Ev. lenti* (Mangabeira, 1938), *Brumptomyia* sp. (França and Parrot, 1921), and *Pressatia* sp. (Mangabeira, 1942). Using PCR with internal transcribed spacer target to identify infected sand flies, five *Lu. longipalpis* females were infected with *Leishmania* spp. Despite the small number of specimens collected, considerable species diversity was found in the study area.

## Introduction

Leishmaniasis is a group of diseases caused by protozoa within the genus *Leishmania* that are transmitted through the bites of female sand flies (1). The transmission pattern of *Leishmania* in Brazil has undergone changes as leishmaniasis is no longer only considered a rural disease as there has been an expansion of the disease throughout Brazil with no signs that the spread is under control (2). There are more than 900 species of sand flies described in the world, 500 of which occur in the Neotropical region (3). Studies addressing the relationships among sand flies, protozoans, and hosts are of fundamental importance to control measures aimed at reducing the expansion of leishmaniasis (4).

Phlebotomine sand flies were only recently described in the city of Lavras in the southern portion of the state of Minas Gerais (southeastern Brazil) after an epidemiological canine serological survey was performed, in which the 579 of 6783 are infected with *Leishmania* sp. In 2017, the first case of human visceral leishmaniasis was detected in the area, which reflects the expansion of *Leishmania* transmission into the urban environment within the country.

Faced with this serious and still neglected public health problem, it is necessary to conduct studies that investigate not only human and canine cases, but also the sand fly fauna, since these insects constitute the vector that maintains the transmission cycle of *Leishmania*. Thus, the aim of the present study was to investigate the sand fly fauna in the municipality of Lavras in the state of Minas Gerais and its natural infection by *Leishmania* spp.

## Material and Methods

### Study area

**Figure 1.**
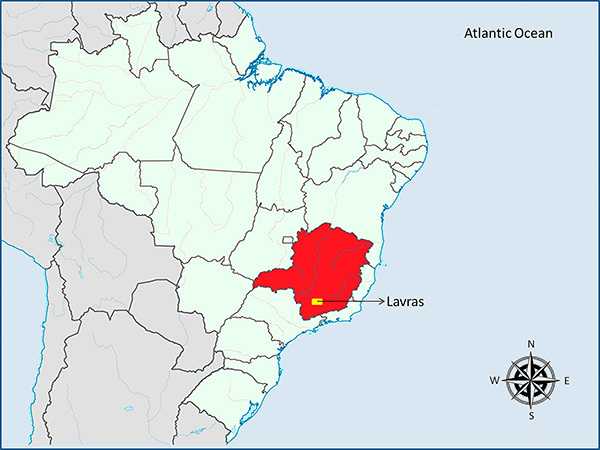
Location of the municipality of Lavras, Minas Gerais, Brazil.

This study was conducted in Lavras (Figure 1), a municipality in the state of Minas Gerais in southeastern Brazil (21°14′43"S 44°59′59"W) located 184 km from the state capital Belo Horizonte. According to the Köppen classification, the climate of the city is Cwa: subtropical dry winter with temperatures below 18°C and hot summer with temperatures above 22°C (5).

### Collection and identification of sandflies

Automatic HP light traps (HP Biomédica, Brazil) (6) were installed from February 2016 to February 2017 in the peridomicile area of residences located in areas with a high incidence of canine visceral leishmaniasis. Six traps were installed (one per residence) at 6:00 p.m. and removed at 6:00 a.m. over three consecutive days, resulting in a sampling effort of 216 total trap hours for this collection effort. Shannon traps were also deployed in semi-preserved areas and HP light traps were installed in the peridomicile area of the residence at which the first human case of visceral leishmaniasis was reported in the area.

Male and female phlebotomine sand flies were identified using the classification proposed by Galati (7). Male sand flies were slide-mounted in Canada balsam. Female sand flies were dissected on a slide containing PBS and then placed in Berlese fluid overnight for clarification (8). All female insects were further identified focusing on the head to view the cibarium and the four final segments in the eighth tergite to view the spermatheca.

### DNA extraction from female sandflies and PCR directed at internal transcribed spacer I (ITS I)

Deoxyribonucleic acid was extracted using the Gentra Puregene kit (Qiagen, USA) following previous protocol (9). The assessment of nucleic acid purity was assessed in a NanoDrop 2000/2000c Spectrophotometer (Thermo Fisher Scientific, USA) using the absorbance ratio of A260/280, and the DNA-concentration was evaluated in Qubit Fluorometric Quantitation (Thermo Fisher Scientific) using DNA Broad Range protocol as described by the manufacturer. PCR samples were heated at 37°C for 20 min. Samples were prepared for a final volume of 25 μL containing 5 μL of the DNA sample to be tested, 1x buffer solution, 1.5 mM of MgCl_2_, 0.2 mM of dNTP mix, and 0.5 μM of each primer (LITSR: 5′CTGGATCATTTTCCGATG3′ and L5.8S: 5′TGATACCACTTATCGCACTT3′) to amplify a fragment of 300 ± 350 bp of the intergenic region of *Leishmania* DNA (internal transcribed spacer q-ITS1) (10). The thermocycling parameters were the same as described in a previous study (11). Amplifications were processed in an Eppendorf¯ Mastercycler Gradient automatic thermal cycler (Germany), subjected to electrophoresis in 1% agarose gel, and stained with ethidium bromide (10 mg/mL). In all reactions, reference strains of *L. braziliensis* (MHOM/BR/75/M2903) were used as positive controls.

## Results

A total of 70 sand fly specimens were collected with the HP light traps in the peridomicile areas and 16 were collected with Shannon's traps in semi-preserved areas (Table 1). Only one of the specimens collected with Shannon's traps was identified (a *Lutzomyia longipalpis* male). Twenty-two female specimens were not identified due to loss of morphological structures.

**Table 1.** Species of sand flies collected in Lavras, Minas Gerais, Brazil.

Species	Male	Female	Relative abundance
*Lutzomyia longipalpis*	22	21	0.67
*Migonemyia migonei*	2	8	0.15
*Evandromyia complex cortelezzii*	1	4	0.07
*Nyssomyia whitmani*	0	2	0.03
*Psathyromyia lutziana*	1	0	0.02
*Evandromyia lenti*	0	1	0.02
*Brumptomyia sp*.	0	1	0.02
*Pressatia sp*.	0	1	0.02
Total	26	38	1.00

From February 2016 to March 2016 (rainy season), HP light traps were installed weekly, resulting in a sampling effort of 1728 h. In all other months, HP light traps were installed only one week per month, resulting in a sampling effort to 216 h per month. The highest number of specimens was found in July (dry season). Shannon's traps were deployed in semi-preserved areas in September, October and December, with the highest number of specimens collected in September (14 sandflies).

The molecular analysis revealed that five (8.33%) out of 60 females of flies captured were positive for *Leishmania* DNA. Positive females were all identified as *Lutzomyia longipalpis*.

## Discussion

Several sand fly species have been implicated as *Leishmania* vectors and usually this association is made according to the dominant species in a given endemic area. Studies on the prevalence of *Leishmania* infection in sand flies are important indicators of the intensity of parasite transmission (12).

The kinetoplast is present in 10,000 copies per cell and its sequence is known in most species of *Leishmania*. Thus, we performed a PCR assay designed to amplify a fragment of 300±350 bp of the intergenic region of *Leishmania* DNA (q-ITS1) due to their good applicability (10). The molecular methods, such as PCR-based techniques, have a high sensitivity and specificity, regardless of the number, stage, and location of the parasite in the insect gut, being an important tool in epidemiological studies to identify infected sand flies and to determine their infection rates in areas endemic for leishmaniasis (13).

With the exception of *Lu. longipalpis*, which was the most abundant species, low relative abundance was found for most sand fly species in Lavras (Table 1). The number of species found corresponds to 7.21% of the 97 species of sandflies registered in the entire state of Minas Gerais (14).

*Lutzomyia longipalpis* is characterized by high adaptability to human environments (15). In Brazil, this is the most important vector involved in the transmission of *Leishmania infantum*, which is responsible for the viscerotropic form of leishmaniasis, a more severe clinical form of the disease (16,17). Other species found also merit attention, such as *Migonemyia migonei,* which is described as a potential vector of *Leishmania infantum*, *Leishmania mexicana*, *Leishmania guyanensis*, and *Leishmania panamensis*; and *Nyssomyia whitmani*, which is a confirmed vector of *Leishmania braziliensis*, *Leishmania guyanensis*, and *Leishmania shawi* as well as a probable vector of *Leishmania lainsoni* (18).

In order to study the presence of *Leishmania* DNA in sand flies in the municipality of São João das Missões in northern Minas Gerais state, the presence of *Leishmania* DNA was detected in eleven samples from peridomicile areas, twelve samples from among the trails (19). In a study on the sand fly fauna in Belo Horizonte city, also in the state of Minas Gerais, a total of 579 phlebotomine sand flies were collected from which 68.7% were females and those specimens were used for natural infection examination by PCR. No *Leishmania* DNA was present in any of the specimens tested (20). The sand fly infection rate in the municipality of Janaúba was 3.9%, according to Michalsky et al. (15). Expansion of the geographic distribution of leishmaniasis has been notified in nearly all states of Brazil, including Minas Gerais where it is endemic. There is considerable species diversity in the municipality studied, where human cases of visceral leishmaniasis are beginning to be reported.
